# Fluorescence Enhancement of Dicyanomethylene-4H-Pyran Derivatives in Solid State for Visualization of Latent Fingerprints

**DOI:** 10.3389/fchem.2022.943925

**Published:** 2022-07-12

**Authors:** Yi Cai, Ting-Ting Hou, Cai-Yun Wang, Ying-Hao Tang, Zhen-Yu Zhang, Deteng Zhang, Ming-Qiang Zhu, Ya-Long Wang

**Affiliations:** ^1^ Key Laboratory of Biomedical Engineering of Hainan Province, School of Biomedical Engineering, Hainan University, Haikou, China; ^2^ Institute of Neuroregeneration and Neurorehabilitation, Qingdao University, Qingdao, China; ^3^ Wuhan National Laboratory for Optoelectronics, Huazhong University of Science and Technology, Wuhan, China; ^4^ One Health Institute, Hainan University, Haikou, China

**Keywords:** latent fingerprints (LFP), fluorescent imaging, dicyanomethylene-4H-pyran (DCM), D-π-A structure, fluorescence enhancement

## Abstract

The efficient development of latent fingerprint (LFP) is attractively important for criminal investigation. The low-cost and high-contrast developer is still a challenge. In this study, we designed and synthesized dicyanomethylene-4H-pyran (DCM) derivatives PZ-DCM and Boc-PZ-DCM by introducing of large steric hindrance group Boc, the solid-state fluorescence of DCM derivatives was greatly enhanced. The low-cost fluorescent LFP developers were prepared by blending with different proportion of montmorillonite (MMT). As a result, clear and high contrast fingerprint patterns were obtained with dusting method by the developer with 3% content of Boc-PZ-DCM. Furthermore, we employed the developer with 3% content of Boc-PZ-DCM to develop the sweat latent fingerprints on different substrates by powder dusting, and collected clear fingerprint patterns, indicating that the developer is universal. In a word, the Boc-PZ-DCM/MMT powder is a promising candidate for LFP developer.

## Introduction

Fingerprint is a complex pattern composed of ridges (raised papillary line) and furrows (sunken wrinkles) through interval distribution (Wang et al., 2017). This unique pattern is different for each person and does not change throughout one’s life (Becue, 2016; Tian et al., 2022). Based on its uniqueness, fingerprint has been used as a person’s ID card for identification in criminal cases (Wang et al., 2017; Wang Y. et al., 2018). Latent fingerprint (LFP), the imprint of a real fingerprint, is crucial evidence for individual identification in forensic science. However, as the most common evidence at a crime scene, LFP is invisible to the naked eye without any treatment. Hence, the efficient visualization of LFP is very important for criminal investigation (Wei et al., 2016; Zhang et al., 2017). Powder dusting, reagent fuming and chemical staining are traditional methods of LFP development (Wang et al., 2017; Li et al., 2020; Lv et al., 2021). The common developers of reagent fuming and chemical staining methods are iodine, cyanoacrylate, silver nitrate, ninhydrin and 1-diazafene-9-one (DFO). These chemical reagents can react with amino acids in LFPs and seriously damage skin, eyes, mucous membrane, and DNA (Wang et al., 2017). Powder dusting method is the most commonly used method due to its simplicity and high efficiency. The tools used are usually special brush and fine powder in contrast to the substrate. When the special brush gently sweeps on the surface of latent fingerprints (LFPs), the powders are adhered to the ridges of latent fingerprints (LFPs), hence the LFPs can be seen by the naked eyes (Li et al., 2020). Traditional powder developers, such as toner, aluminum, copper, magnetic powder, are not suitable for substrates with similar color and often show low signal-to-noise ratio and low resolution. Hence, high contrast powder developer is an urgent problem to be solved. Fluorescent materials have been widely studied due to their various colors, high brightness and high contrast. Fluorescence visualization of LFPs has become a new research hotspot (Wang et al., 2020). Up to now, a variety of fluorescent materials have been employed in LFPs fluorescence development, such as up-conversion nanoparticles (UCNPs) (Wang et al., 2015; Marappa et al., 2018; Kanodarwala et al., 2021), metal−organic frame-works (Wang M. et al., 2018), quantum dots (QDs) (Raju et al., 2017; Sandhyarani et al., 2017; Shi et al., 2018), gold nanoparticles (Cheng et al., 2016), carbon dots (Fernandes et al., 2015), and semiconductor polymer dots (Cui et al., 2015). Compared with traditional developers, fluorescent developers greatly improve the contrast of development patterns of LFPs. However, these fluorescent materials showed some limitations, such as complex preparation process, large consumption of developers, exorbitant price, poor safety and so on (Wang et al., 2020). Recently, Pro Fu and Pro Tang prepared LFP fluorescent developer by blending fluorescent dyes with MMT (Li et al., 2020; Lv et al., 2021). These novel fluorescent developers reduced the dye content to 10%, which greatly reduced the consumption of developers and enhanced the safety. At present, there are few studies on fluorescent dye/MTT developers. Novel fluorescent materials for fluorescent dye/MTT developers with lower material consumption and high performance of LFPs development need to be developed.

As one of the typical fluorophores, DCM derivatives possess excellent photophysical and photochemical properties, such as large Stokes shift, excellent photostability, tunable near-infrared emission, etc. However, the fluorescence of DCM derivatives in solid state is usually very weak, suffering from aggregation-caused quenching (ACQ) effect. Herein, DCM derivative Boc-PZ-DCM with strong emission in solid state was successfully synthesized by introducing Boc group into the core of DCM. By blending Boc-PZ-DCM with MMT, fluorescence developer for LFP was successfully prepared, reducing the dosage of fluorescent material to 3%. This paper provides a strategy for fluorescence enhancement of dyes in solid state and its application in efficient fluorescence visualization of LFP.

## Materials and Methods

Synthesis of (*E*)-2-(2-methyl-6-(4-(piperazin-1-yl)styryl)-4H-pyran-4-ylidene)malononitrile (PZ-DCM). A solution of 2-(2,6-dimethyl-4H-pyran-4-ylidene)malononitrile (200 mg, 1.16 mmol, 1 eq.) and 4-(piperazin-1-yl)benzaldehyde (221 mg, 1.16 mmol, 1 eq.) and piperidine (20 μl, cat.) in EtOH (15 ml) was stirred at 80°C for 16 h. The solvent was evaporated under vacuum. The residue was purified by flash column chromatography (DCM/MeOH = 0–15%) to afford **PZ-DCM** (160 mg, 40%) as an orange solid. ^1^H NMR (400 MHz, Chloroform-*d*_*3*_) δ 7.46–7.42 (m, 2H), 7.37 (d, J = 15.9 Hz, 1H), 6.91 (d, J = 8.9 Hz, 2H), 6.61 (d, J = 2.1 Hz, 1H), 6.56–6.47 (m, 2H), 3.33–3.23 (m, 4H), 3.07–2.97 (m, 4H), 2.39 (s, 3H). ^13^C NMR (101 MHz, DMSO-*d*_6_) δ 164.32, 161.29, 157.14, 153.05, 138.53, 130.08, 124.75, 116.22, 114.74, 114.70, 106.04, 105.99, 48.35, 45.78, 19.86.

Synthesis of tert-butyl (*E*)-4-(4-(2-(4-(dicyanomethylene)-6-methyl-4H-pyran-2-yl)vinyl)phenyl)piperazine-1-carboxylate (Boc-PZ-DCM). A solution of 2-(2,6-dimethyl-4H- pyran-4-ylidene)malononitrile (500 mg, 2.91 mmol, 1 eq.) and tert-butyl 4-(4-formylphenyl)piperazine-1-carboxylate (844 mg, 2.91 mmol, 1 eq.) and piperidine (40 μl, cat.) in EtOH (30 ml) was stirred at 80°C for 16 h. The solvent was evaporated under vacuum. The residue was purified by flash column chromatography (DCM/MeOH = 0–10%) to afford **Boc-PZ-DCM** (810 mg, 63%) as a red solid. ^1^H NMR (600 MHz, Chloroform-*d*_*3*_) δ 7.46–7.42 (m, 2H), 7.37 (d, J = 15.9 Hz, 1H), 6.91 (dd, J = 8.7, 5.9 Hz, 2H), 6.62 (d, J = 2.1 Hz, 1H), 6.57–6.48 (m, 2H), 3.59 (t, J = 5.4 Hz, 4H), 3.29 (t, J = 5.2 Hz, 4H), 2.36 (s, 3H), 1.49 (s, 9H). ^13^C NMR (101 MHz, DMSO-*d*_6_) δ 164.37, 161.19, 157.16, 154.33, 138.37, 131.96, 130.06, 126.90, 125.30, 116.18, 115.23, 113.88, 106.58, 106.14, 79.55, 47.30, 46.61, 28.53, 19.86, 19.78.

Preparation of Boc-PZ-DCM/MMT developers. Boc-PZ-DCM/MMT developers were prepared by grinding Boc-PZ-DCM and MMT with a mortar. 10 mg Boc-PZ-DCM and 1990 mg MMT were thoroughly ground to prepared 0.5% Boc-PZ-DCM/MMT developer. 1%, 3%, 5% Boc-PZ-DCM/MMT developers were prepared by grinding 20 mg Boc-PZ-DCM and 1980 mg MMT, 60 mg Boc-PZ-DCM and 1940 mg MMT, 100 mg Boc-PZ-DCM and 1900 mg MMT, respectively.

## Results and Discussion

**FIGURE 1:**
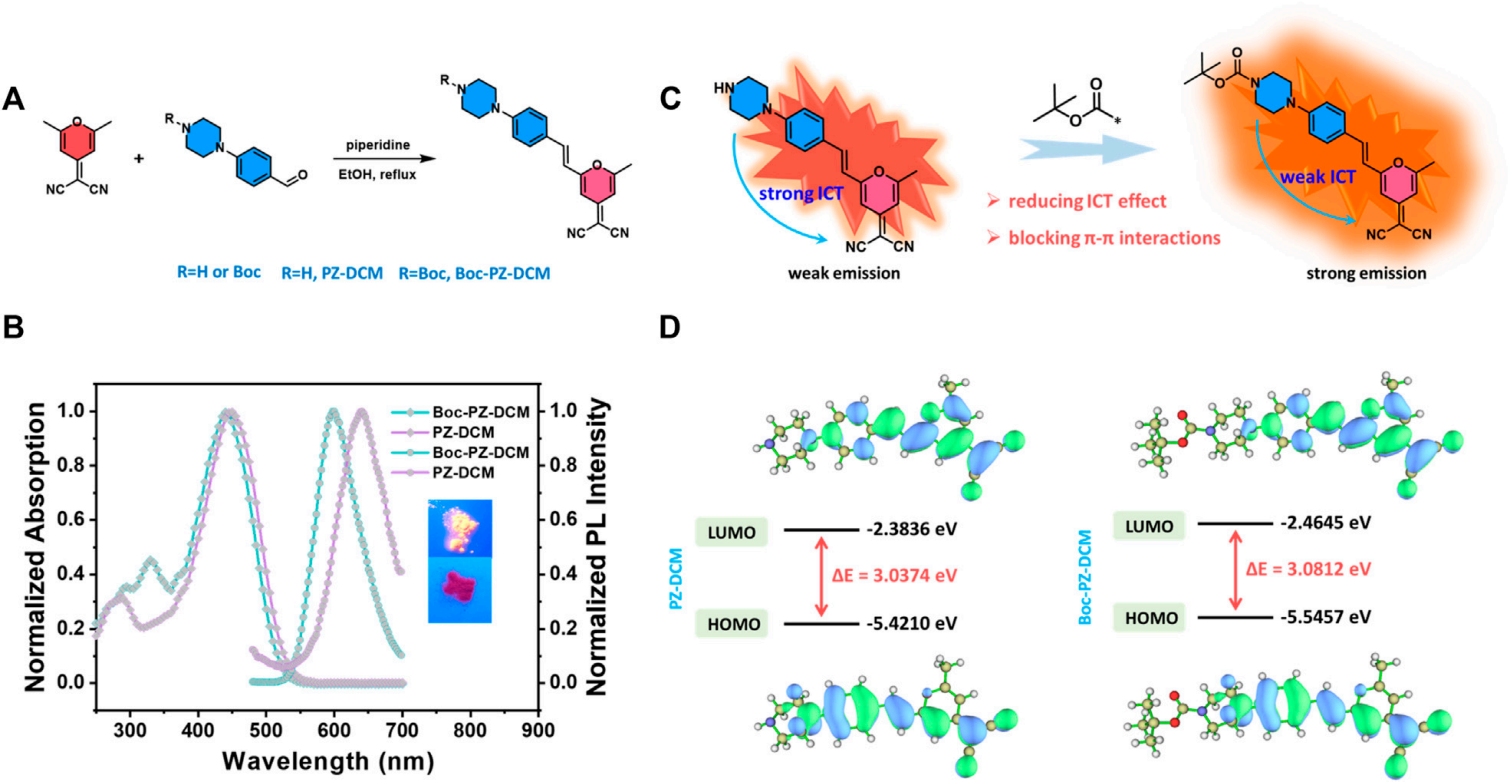
**(A)** The synthetic route of compounds PZ-DCM and Boc-PZ-DCM. **(B)** Normalized absorption and fluorescence spectra of PZ-DCM and Boc-PZ-DCM. The absorption spectra were measured in THF solution with the dye concentration of 10 μM. The fluorescence spectra were measured in solid state under 410 nm excitation. Inset: Fluorescent photographs of PZ-DCM (bottom) and Boc-PZ-DCM (top) under 365 nm irradiation. **(C)** Schematic diagram for fluorescence properties of PZ-DCM and Boc-PZ-DCMS affect by ICT effect. **(D)** HOMO and LUMO energy levels of PZ-DCM and Boc-PZ-DCM. Molecular orbital amplitude plots of HOMO and LUMO energy levels calculated using the B3LYP/6-31G(d) basis set in the Gaussian 09 program. ∆E (energy gap) = LUMO–HOMO.

PZ-DCM and Boc-PZ-DCM were synthesized by simple Knoevenagel condensation (Figure 1A). The detailed synthesis procedures of PZ-DCM and Boc-PZ-DCM and the target compounds are described in section of materials and methods. The target compounds were characterized by ^1^H NMR, ^13^C NMR. The UV-vis absorption spectra of PZ-DCM and Boc-PZ-DCM were measured in THF solution. The maximum UV-vis absorption peaks of PZ-DCM and Boc-PZ-DCM are located at 450 and 440 nm, respectively (Figure 1B). Compared with PZ-DCM, the maximum absorption peak of Boc-PZ-DCM blue-shifted 10 nm. This is probably attributed to the introduction of the Boc electron-withdrawing group. The photoluminescence (PL) spectra of PZ-DCM in solid state located at 640 nm and the solid of PZ-DCM showed weak red emission (Figure 1B). However, Boc-PZ-DCM emitted strong orange fluorescence in solid state and the emission peak located at 598 nm (Figure 1B). The emission peak showed a large blue-shift of 42 nm for Boc-PZ-DCM compared with PZ-DCM in solid. This is mainly attributed to the electron-withdrawing effect and steric effect of Boc group. To understand their geometries and electronic structures at the molecular level, density functional theory (DFT) calculations of PZ-DCM and Boc-PZ-DCM were carried out (Lu and Chen, 2012). The optimized geometries of PZ-DCM and Boc-PZ-DCM are shown in Figure 1D. The computerized energy gap (∆Eg) of PZ-DCM and Boc-PZ-DCM are 3.0374 and 3.0812 eV, respectively. Combined with the calculation results, it can be inferred that, firstly, the electron-withdrawing effect of Boc group reduces the electron donating capacity of the piperazine group, hence, the ICT effect of Boc-PZ-DCM is weakened (Figures 1C,D). Secondly, the large steric hindrance of the Boc group prevents strong intermolecular π-π stacking of Boc-PZ-DCM. This means the Boc group can greatly affect the luminescence properties of DCM derivatives.

**FIGURE 2:**
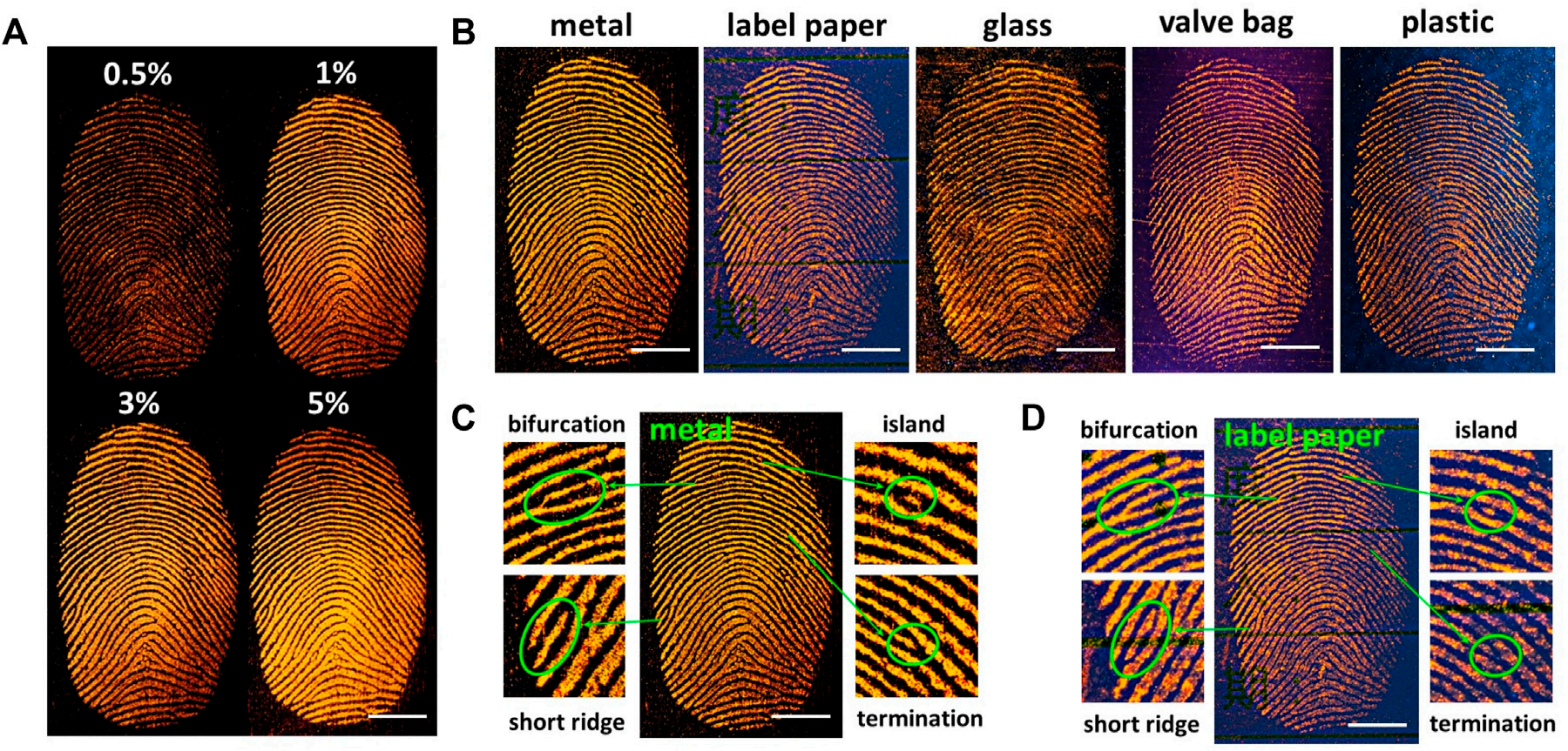
**(A)** Images of LFPs developed by the mixing powder of Boc-PZ-DCM/MMT with dye content of 0.5%, 1%, 3%, 5%, respectively, under 365 nm irradiation. **(B)** Images of LFPs on different substrates developed by the mixing powder of Boc-PZ-DCM/MMT with dye content of 3%. **(C)** Level 2 details of LFP on metal developed by the mixing powder of Boc-PZ-DCM/MMT with dye content of 3%. **(D)** Level 2 details of LFP on labelled paper developed by the mixing powder of Boc-PZ-DCM/MMT with dye content of 3%. Scale bar, 5 mm.

Fluorescent materials have been used for LFP visualization with powder dusting method due to its high contrast and low background. However, the traditional fluorescent developer is high consumption in LFP visualization. Recently Tang et al. (Li et al., 2020) and Fu et al. (Lv et al., 2021) prepared LFP fluorescent developer by blending fluorescent materials with MMT. This method reduces the use of fluorescent materials to 10%. The excellent optical performance of Boc-PZ-DCM is conducive to the application of LFPs fluorescent development. Hence, Boc-PZ-DCM/MMT fluorescent developers were prepared by mixing Boc-PZ-DCM and MMT with dye content of 0.5%, 1%, 3%, 5%, respectively. Next, the LFPs were developed by dusting method with the prepared fluorescent developers. As shown, when the dye content was 0.5%, the developed fingerprint pattern showed poor contrast (Figure 2A). When the dye ratio was between 1% and 5%, the fingerprint pattern was clearly visible, showing excellent contrast (Figure 2A). Subsequently, Boc-PZ-DCM/MMT developer with 3% dye content was employed to develop LFPs on different substrates (Figure 2B). Clear fingerprint patterns can be obtained with high resolution. Level 2 details, generally divided into termination, bifurcation, island, short ridge, lake and so on, are often used to recognize an individual’s identity due to the unique features of individual. To verify the fingerprint details, level 2 details of the fingerprints on metal and label paper were analyzed. As shown, the bifurcation, short ridge, island and termination are clearly visible by the naked eye, which can provide reliable evidence for the reorganization of individual identity (Figures 2C,D). The results show that Boc-PZ-DCM/MMT developer possesses high development ability and multi-substrates universality.

## Conclusion

In conclusion, we have successfully prepared DCM derivatives with strong emission in solid state by introducing Boc group, which provides strategies for the fluorescence enhancement of ACQ dyes in solid state. In view of this, LFP fluorescent developers were prepared by blending Boc-PZ-DCM with MMT. As a result, LFP can be clearly developed by dusting method with 3% dye content Boc-PZ-DCM/MMT developer. The level 2 fingerprint features are clearly visible, resulting in a clear, high-contrast fingerprint pattern. Boc-PZ-DCM/MMT developer for LFP reduces the content of fluorescent materials to 3%, greatly reducing the consumption of fluorescent materials and increasing the safety of LFP fluorescent developer.

## Data Availability

The original contributions presented in the study are included in the article/Supplementary Material, further inquiries can be directed to the corresponding author.
